# Comparison of Dexmedetomidine Versus Propofol in Mechanically Ventilated Patients With Sepsis: A Meta-Analysis of Randomized Controlled Trials

**DOI:** 10.3389/fphar.2022.901898

**Published:** 2022-05-26

**Authors:** Hua-Ze Ding, Yi-Ling Dong, Kai-Yue Zhang, Jia-Yu Bai

**Affiliations:** School of the First Clinical Medical Sciences, Wenzhou Medical University, Wenzhou, China

**Keywords:** dexmedetomidine, propofol, sepsis, sedation, mechanical ventilation

## Abstract

**Purpose:** The aim of the present study was to evaluate the effects of dexmedetomidine compared with propofol in mechanically ventilated patients with sepsis.

**Methods:** We searched PubMed, EMBASE, and Cochrane Library for randomized controlled trials comparing the effects of dexmedetomidine versus propofol in septic patients requiring mechanical ventilation from inception to December 2021. The primary outcome was 28/30-day mortality and secondary outcomes were ventilator-free days and the length of ICU stay. Pooled relative risk (RR), mean deviation (MD), along with 95% confidence intervals (CI) were used to express outcomes by the software of Review Manager 5.3.

**Results:** Seven studies with a total of 1,212 patients were eligible for meta-analysis. The results primarily showed that dexmedetomidine had no significant effects on the 28/30-day mortality (RR = 1.04 [0.85–1.26], *p* = 0.70, I^2^ = 3%). As for secondary outcomes, the administration of dexmedetomidine was not associated with longer-ventilator-free days (MD = 0.50 [−2.15, 3.15], *p* = 0.71, I^2^ = 24%) compared with propofol. However, our results revealed dexmedetomidine could shorten the length of ICU stay (MD = −0.76 [−1.34, −0.18], *p* = 0.01, I^2^ = 33%).

**Conclusion:** Administration of dexmedetomidine for sedation in septic patients who required mechanical ventilation had no effect on 28/30-day mortality and ventilator-free days, but it could shorten the length of ICU stay.

## 1 Introduction

Sepsis, which is defined as life-threatening organ dysfunction caused by a dysregulated host response to infection, contributes the highest mortality to intensive care units (ICU) worldwide (Mayr et al., 2014; Fleischmann et al., 2016). Because of the high incidence of respiratory failure in sepsis care, mechanical ventilation is always adopted to give life support and minimize lung injury (Dellinger et al., 2012). And sedation is a necessary component of sepsis care who suffers from mechanical ventilation (Reade and Finfer, 2014). The Society of Critical Care Medicine (Devlin et al., 2018) suggested using either propofol or dexmedetomidine for sedation in mechanically ventilated adults. However, it remained unknown whether patients with sepsis requiring mechanical ventilation will benefit from sedation with dexmedetomidine.

Dexmedetomidine (DEX), a potential alternative sedative, is a high-affinity α2 agonist (Keating, 2015). Recently, DEX has been proved to attenuate sepsis-associated inflammation (Mei et al., 2021) and reduce ventilator-induced lung injury (Zhu et al., 2020) by animal experiments, which seems to be superior to the traditional sedatives, such as propofol and benzodiazepines. However, a recent large-scale RCT reached a conclusion that among patients undergoing mechanical ventilation in the ICU, those who received early dexmedetomidine for sedation had a similar rate of 90-day mortality compared with the propofol group (Shehabi et al., 2019). The effects of two agents on septic patients remained controversial. Several randomized controlled trials (RCTs) have compared dexmedetomidine with propofol for sedation in septic patients in some respects, but drawn different conclusions. Liu et al. (Liu et al., 2020) concluded that dexmedetomidine could significantly reduce the length of ICU stay among septic patients undergoing mechanical ventilation, while a noninferiority trial comparing dexmedetomidine with propofol in critically ill patients, about half of whom had sepsis, showed that the choice of sedation didn’t affect the length of stay in ICU and short-term mortality (Jakob et al., 2012). A multicenter and double-blind RCT organized by Hughes et al. (Hughes et al., 2021) also declared that there was no significant difference in short-term mortality, ventilator-free days and ICU length of stay between septic adults who received dexmedetomidine or propofol.

Due to the controversy, several meta-analyses have been conducted to evaluate the efficacy of DEX in mechanically ventilated patients with sepsis last year. Although these meta-analyses draw the similar primary outcome, several limitations existed indeed. Huang et al. (Huang et al., 2021) involved fifteen trials, but the majority of their trials were published in Chinese and only four studies were published in English. It was believed that the language would limit the extrapolation of results and it was hard to guarantee the quality of research. The largest sample size in the rest previous meta-analysis that explicitly adopted propofol as a comparator was 933 patients (Wang et al., 2021) and there were only four trials that reported the short-term mortality. Liu et al. (Liu et al., 2022) reported that DEX had no effect on length of ICU stay. However, they only involved five RCTs and their comparison included lorazepam and midazolam. The meta-analysis implemented by Abdelazeem et al. (Abdelazeem et al., 2022) was also constrained by the same limitations, which might cause inaccurate results.

Therefore, our present meta-analysis, setting propofol principally for the control group and including a bigger number of participants, aims to determine whether sedation with DEX affected the short-term mortality, ventilator-free days and the length of ICU stay in septic patients.

## 2 Methods

### 2.1 Protocol and Registration

The present research protocol was registered at INPLASY (Registration NO: INPLASY202240103) (https://inplasy.com/), following a prespecified protocol and complying with the Preferred Reporting Items for Systematic Meta-Analyses.

(PRISMA) guideline (Page et al., 2021). Therefore, our meta-analysis was conducted based on PRISMA guideline, as recommended by the Cochrane Collaboration.

### 2.2 Search Strategy

We systematically searched EMBASE, PubMed (MEDLINE), Cochrane Library from inception to December 2021. No language restrictions were imposed. We used the following combined text and Mesh terms: “sepsis” and “dexmedetomidine” in PubMed. The complete search strategies were shown in **Supplemental file 1.** In addition, Clinical.gov was searched for ongoing studies and unpublished data. A hand search through relevant conference papers and reference lists of relevant articles or reviews was also performed for completeness.

### 2.3 Eligibility Criteria

Inclusion criteria:

1) Study design: Randomized controlled trials (RCTs).
2) Participants: All patients were diagnosed as sepsis and required mechanical ventilation.
3) Intervention: dexmedetomidine with or without other sedatives, irrespective of dose and duration.
4) Comparison: propofol without dexmedetomidine, irrespective of dose and duration.
5) Outcomes: the primary outcome: 28/30-day mortality.
  the secondary outcomes: ventilator-free days and the length of ICU stay.

Exclusion criteria: pediatrics, duplicated data, reviews, commentaries, meeting abstract, meta-analyses, animal and cell experiments, no clear diagnosis of sepsis.

### 2.4 Study Selection and Data Extraction

Two review authors independently screened the literature, extracted data, and cross-checked each other. First, duplicated articles were excluded. Then, irrelevant articles were excluded after screening titles and abstracts. Finally, we determined included studies after reading the full text of the remaining studies.

The following data were extracted: the first author’s name, publication year, sample size, country of study, characteristics of included patients such as age, APACHE II score and sedation levels, strategies of intervention and control group, outcomes, and other items necessary for quality evaluation. We also contacted corresponding authors for completed data and risk of bias ratings. Disagreement during the review process was resolved by consensus or involving a third review author.

### 2.5 Risk of Bias Assessment

The Cochrane Collaboration’s tool (Higgins et al., 2011) was used to assess the qualities of included studies by two authors assessed the qualities of all eligible studies in Review Manager 5.3 (Cochrane Collaboration, Oxford, United Kingdom), which contains seven aspects: allocation concealment, random sequence generation, blinding of outcome assessment, blinding of participants and personnel, selective reporting, incomplete outcome data, and other bias. Each item was assessed as high risk, uncertain risk, or low risk.

### 2.6 Statistical Analysis

We assessed the effect of dexmedetomidine on three outcomes:28/30-day mortality, ventilator-free days, and the length of ICU stay. The statistical data analyses were performed by the software Review Manager 5.3. Since that some studies (Tasdogan et al., 2009; Kawazoe et al., 2017; Sigler et al., 2018; Liu et al., 2020; Hughes et al., 2021) described the data by median and interquartile range, we asked first and corresponding authors for raw data by email but failed, so we adopted the suggestions of Luo et al. (Luo et al., 2018) and Wan et al. (Wan et al., 2014) to estimate the mean values and standard deviation.

Pooled risk ratio (RR) along with 95% confidence intervals (CI) were used to express the primary outcome, 28/30-day mortality, while for secondary outcomes including ventilator-free days and the length of ICU stay, mean difference (MD) with 95% CI were calculated. The heterogeneity was evaluated using the Chi-square test and Higgins I^2^ test (Higgins et al., 2003); the fixed-effect model was used when I2 ≤ 50% and *p* ≥ 0.10; otherwise, we applied the random effect model to describe the heterogeneity. Besides, the sensitivity analysis was involved to omit one study and assess whether the other results were substantially affected. We designed the sensitivity analysis of 28/30-day mortality to test the robustness of the primary outcome by STATA 15.0(Stata Corp, College Station, TX).

## 3 Results

### 3.1 Literature Search

**FIGURE 1:**
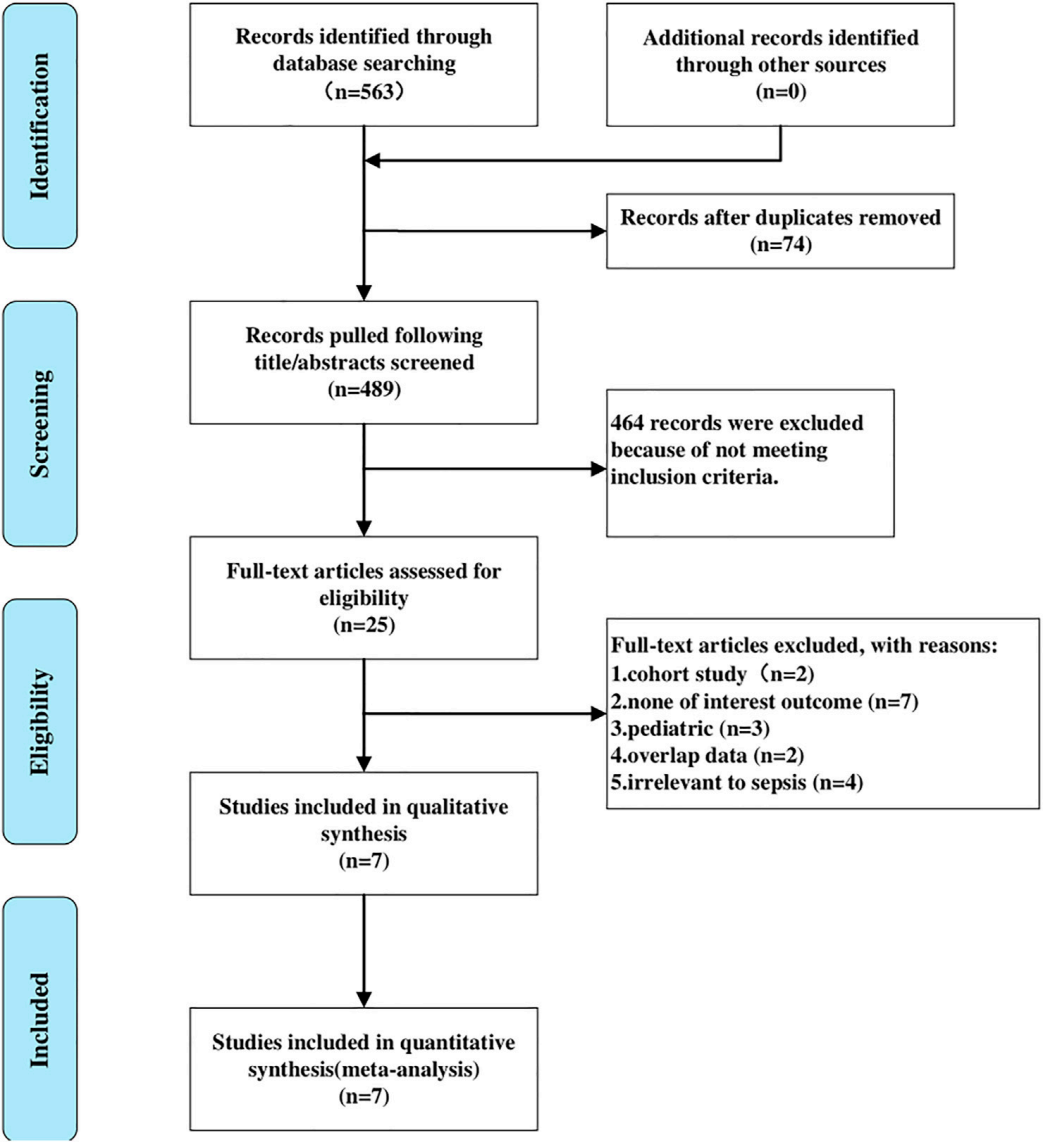
Flow diagram of literature screening and selection process.

In accordance with the search strategy, we yielded 24 titles in Cochrane, 117 in PubMed, and 422 in Embase. A total of 563 relevant records were retrieved in the initial search. After screening titles and abstracts, we removed 74 articles due to duplicates, and 25 articles were selected for full-text reading. Eventually, seven RCTs (Tasdogan et al., 2009; Guo et al., 2016; Kawazoe et al., 2017; Sigler et al., 2018; Ding et al., 2019; Liu et al., 2020; Hughes et al., 2021) meeting the inclusion criteria and passing the following assessment of quality were included in the present meta-analysis. Figure 1 showed the detailed process of literature selection.

### 3.2 Characteristics and Risk of Bias in Included Studies

Seven studies with 596 septic patients in the DEX group and 616 septic patients in the propofol group were eligible. Among seven trials, one was published in Chinese, and six were published in English. Two studies were double-blind (Liu et al., 2020; Hughes et al., 2021), one study is blinded-endpoint (Kawazoe et al., 2017), while others were not blind (Tasdogan et al., 2009; Guo et al., 2016; Sigler et al., 2018; Ding et al., 2019). Detailed information is showed in Table.1.

**TABLE 1:** Characteristics of the included studies.

Study	Age	Country	APACHEII Score	Sedation Levels	Sample Size EG/CG	Type	Patients	Outcomes	Intervention Group	Control Group
Hughes et al. (2021)	EG:59 (48–68) CG:60 (50–68)	USA	EG:27 (21–32) CG:27 (22–32)	RASS score:0 to −2	214/208	Multicenter Double-blind	Sepsis	(1)(2)(3)	DEX 0.2 to 1.5 μg per kilogram of body weight per hour	PRO 5 to 50 μg per kilogram per minute
Tasdogan et al. (2009)	EG:58 (21–78) CG:50 (19–74)	Turkey	EG:18 ± 4 CG:19 ± 5	Ramsay score <2	20/20	Single-center Not blind	Severe sepsis	(1)(3)	DEX one μg/kg over 10 min followed by a maintenance dose of 0.2–2.5 μg/kg/h over 24 h	PRO one mg/kg over 15 min followed by a maintenance dose of one to three mg/kg/hr over 24 h
Kawazoe et al. (2017)	EG:68 ± 14.9 CG:69 ± 13.6	Japan	EG:23 (18–9) CG:22 (16–29.5)	RASS score Day:0 Night:−2	100/101	Multicenter Blinded-endpoint	Sepsis	(1)(2)(3)	DEX + PRO + Midazolam DEX started from 0.1 μg/kg/hr, titrated 0.1–0.7 μg/kg/hr minimum propofol/midazolam as needed	PRO + Midazolam. PRO titrated 0–3 mg/kg/hr Midazolam titrated 0–0.15 mg/kg/hr
Ding et al. (2019)	EG:55.09 ± 10.84 CG:55.27 ± 11.26	China	EG:18.87 ± 4.30 CG:18.67 ± 4.26	NC	131/152	Single-center Not blind	Sepsis	(3)	DEX started from 1 μg/kg/h for 10 min, titrated 0.2–0.7 μg/kg/h.	PRO titrated 0.05 mg/kg/min
Guo et al. (2016)	EG:54.9 ± 20.7 CG:58.2 ± 19.1	China	EG:24.1 ± 4.0 CG:22.5 ± 4.5	RASS score −1 to −2	14/16	Single-center Not blind	Septic shock	(1)(3)	DEX titrated 0.2–0.7 μg/kg/h+propofol	PRO
Sigler et al. (2018)	EG:62.5 ± 9.6 CG:59.0 ± 15.4	USA	EG:19 (13,20) CG:16 (12,19)	RASS score −1 to 1	17/19	Single-center Not blind	Sepsis	(1)(3)	DEX started from 0.2 μg/kg/h and titrated 0.1 μg/kg/hour	PRO started from 5 μg/kg/minute and titrated 5 μg/kg/minute
Liu et al. (2020)	EG:57 [31–66] CG:54 [35–71]	China	EG:29 [26–37] CG:29 [22–36]	RASS score −2 to 0	100/100	Single-center Double blind	Septic shock	(1)(3)	DEX 1 μg/kg over 10 min followed by a maintenance at 0.2–0.3 μg/kg/h for 5 d	PRO 1 mg/kg for 10 min followed by a maintenance at 1–3 mg/kg/h for 5 d

Abbreviations: EG, experimental group; CG, control group; DEX, dexmedetomidine; PRO, propofol; RASS, Richmond Agitation–Sedation Scale

Outcome measures: (1)28/30-day mortality; (2) ventilator-free days; (3) the length of ICU stay.

**FIGURE 2:**
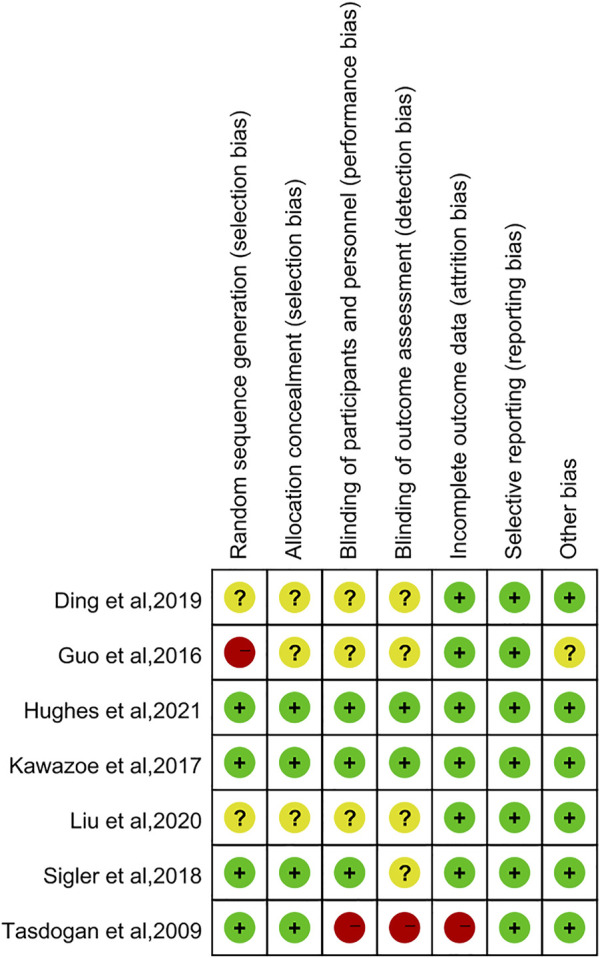
Risk of bias summary.

**FIGURE 3:**
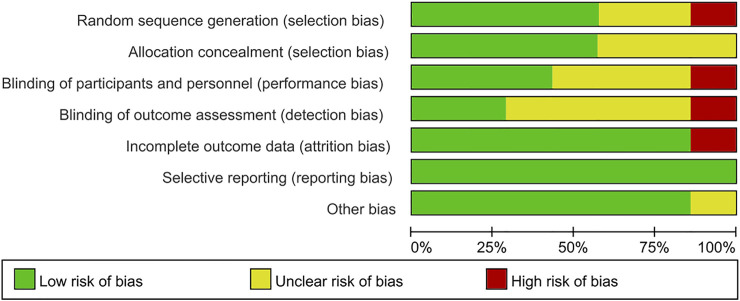
Risk of bias graph.

The Cochrane Collaboration’s tool was used to assess the methodological quality of the seven RCTs. Figures 2, 3 showed summaries of the risk of bias. Overall, the quality of literature was “low risk”.

### 3.3 The Primary Outcome

#### 3.3.1 28/30-Day Mortality

**FIGURE 4:**
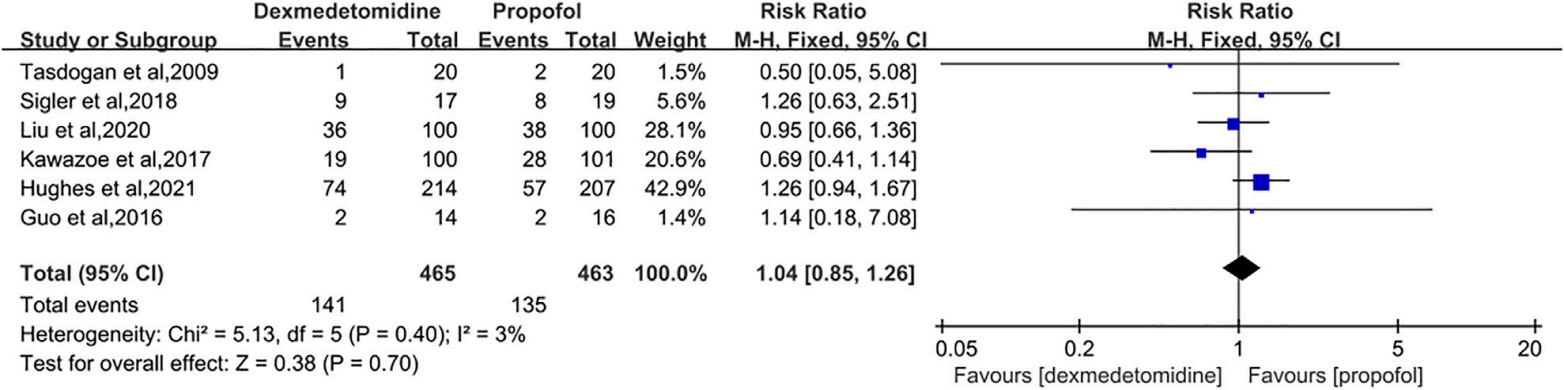
Forest plot comparing the 28/30-day mortality between two groups.

Six studies involving 928 participants were included for the analysis of 28/30-day mortality (Tasdogan et al., 2009; Guo et al., 2016; Kawazoe et al., 2017; Sigler et al., 2018; Liu et al., 2020; Nakashima et al., 2020). No significant heterogeneity between studies was found and the fixed-effects model was applied. The pooling results showed that compared to the propofol group, patients who were given DEX had a similar risk of death in 28/30 days (RR = 1.04 [0.85–1.26], *p* = 0.70, I^2^ = 3%, Figure 4).

### 3.4 The Secondary Outcomes

#### 3.4.1 Ventilator-Free Days

**FIGURE 5:**
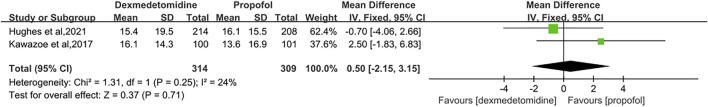
Forest plot comparing ventilator-free days between two groups.

There were two trials measuring the duration of ventilator-free days after treatment (Kawazoe et al., 2017; Hughes et al., 2021). These trials concluded that there was no significant difference between the DEX group and the propofol group. Heterogeneity among the two studies was low, so we adopted a fixed-effect model. Our meta-analysis signified that compared with propofol sedation, dexmedetomidine didn’t shorten the ventilator-free days (MD = 0.50 [−2.15, 3.15], *p* = 0.71, I^2^ = 24%, Figure 5).

#### 3.4.2 Length of ICU Stay

**FIGURE 6:**
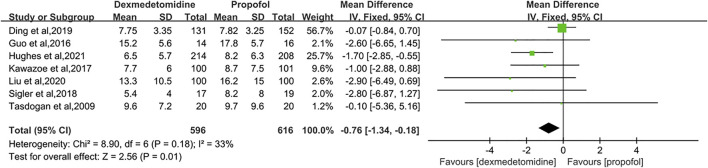
Forest plot comparing the length of ICU stay between two groups.

Seven RCTs reported the length of ICU stay with 1,212 patients enrolled (Tasdogan et al., 2009; Guo et al., 2016; Kawazoe et al., 2017; Sigler et al., 2018; Ding et al., 2019; Liu et al., 2020; Hughes et al., 2021). Because of the moderate heterogeneity among the studies (*p* = 0.18, I2 = 33%), we selected the fixed-effect model to describe this outcome. The result indicated that the DEX group had an advantage over the propofol group in reducing the length of ICU stay by 0.76 days (MD = −0.76 [−1.34, −0.18], *p* = 0.01, I^2^ = 33%, Figure 6).

### 3.5 Publication Bias

As there not enough studies (<10), we were unable to assess the publication bias by using a funnel plot. In general, trials with positive results are more likely to be published than those with negative or neutral results, so we couldn’t rule out the possibility of publication bias.

### 3.6 Sensitivity Analysis

**FIGURE 7:**
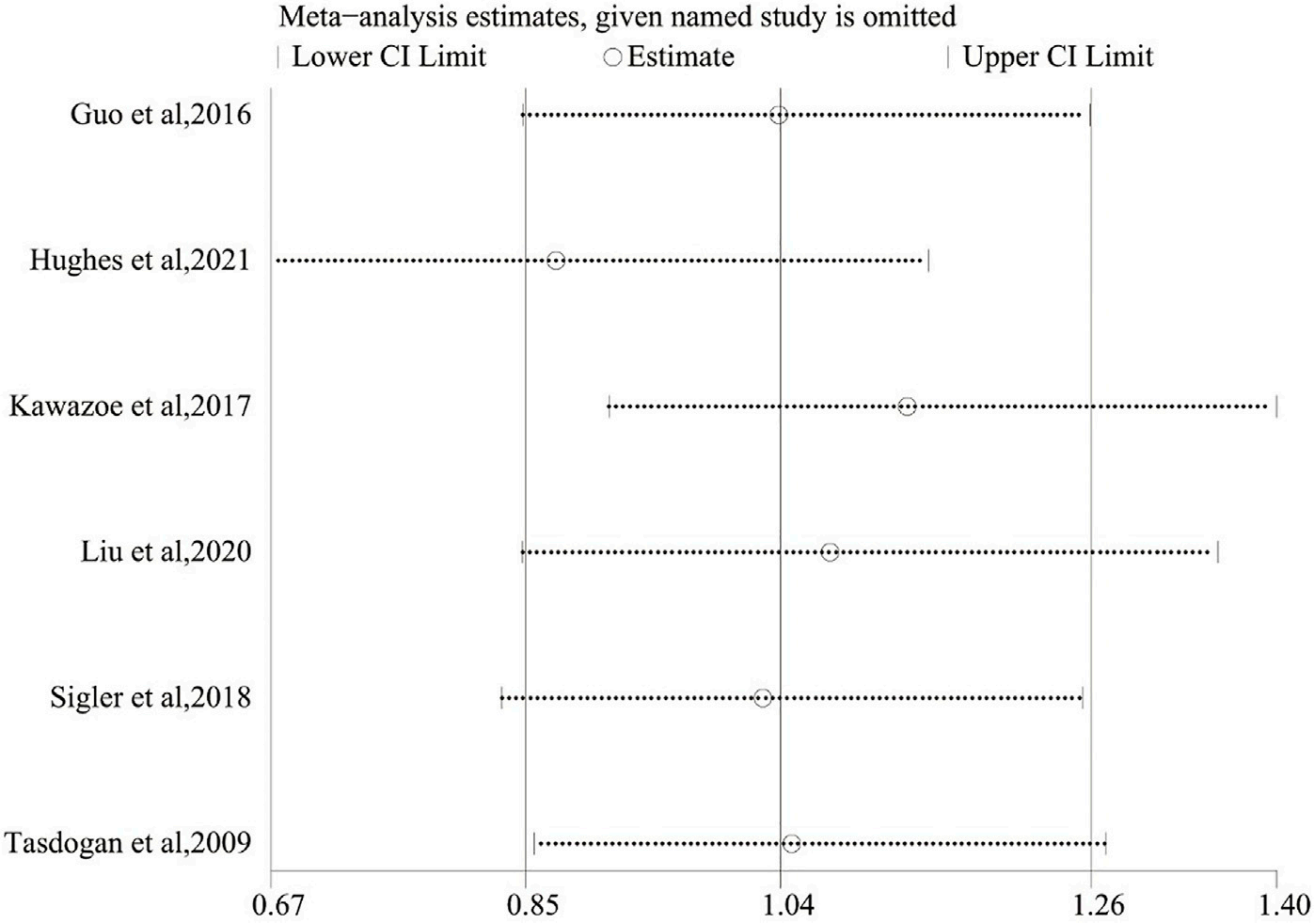
Sensitivity analysis of 28/30-day mortality by STATA.

For the purpose of evaluating the stability of the outcome, a sensitivity analysis of the 28/30-day mortality was performed, which was conducted by sequentially omitting each included study and checking the consistency of the overall effect estimate. Figure 7 revealed that these results were similar, which showed good stability.

## 4 Discussion

In this meta-analysis, our results showed that there was no difference in 28/30-day mortality and ventilator-free days between the dexmedetomidine and the propofol group in mechanically ventilated patients with sepsis. Furthermore, we found the patients who received dexmedetomidine had shorter stays in the ICU compared with those who were treated with propofol.

Our outcomes were different from that of several similar previous meta-analyses (Zhang et al., 2019; Chen et al., 2020; Huang et al., 2021; Wang et al., 2021; Abdelazeem et al., 2022; Liu et al., 2022). Chen et al. (Chen et al., 2020) reported that DEX increased the number of ventilator-free days and reduced 28-day mortality for sedation among mechanically ventilated adult sepsis or septic shock patients, and Zhang et al. (Zhang et al., 2019) concluded that dexmedetomidine could dramatically reduce 28-day mortality. While Chen et al. (Chen et al., 2020) included only four trials involving 349 septic patients and Zhang et al. (Zhang et al., 2019) included 586 patients, we believed that their studies were limited by the small sample size. Furthermore, their interventions of the control group were broad, including sedatives such as midazolam and lorazepam. Although four meta-analyses (Huang et al., 2021; Wang et al., 2021; Abdelazeem et al., 2022; Liu et al., 2022) draw the similar primary outcome as we were in the previous year, we believed that our present study was more persuasive than those because of the larger sample size and the more specific control group. As the rigorous eligibility criteria we designed, our present meta-analysis, including 1,212 patients and setting propofol intervention principally for the control group, rendered the results more convincing.

Results showed that there was no reduction in 28/30-day mortality between the dexmedetomidine group and propofol group among septic patients requiring mechanical ventilation. Aso et al. (Aso et al., 2020) found the dexmedetomidine group had significantly lower 28-day mortality compared with the group receiving midazolam or propofol. However it was a retrospective cohort study without the subgroup analysis of midazolam group and propofol group. In our meta-analysis, we focused on the effect of dexmedetomidine compared with propofol in septic patients. In other words, the intervention of the control group was exclusively propofol in our meta. Two large-scale RCTs reported by Jakob et al. (2012) revealed that whether DEX group compared to propofol group or midazolam group, the 45-day mortality was similar among ICU patients, and Riker et al. (2009) reached similar results, which suggested dexmedetomidine may not reduce mortality compared with traditional sedatives. The trial conducted by Hughes et al. (2021) showed dexmedetomidine didn’t lead to lower 90-day mortality than propofol, thus the long-term mortality of DEX needed to be considered. As for ventilator-free days, this concept results from the combinations of death rates and mechanical ventilation durations (Bodet-Contentin et al., 2018). And ventilator-free days are now widely used as an outcome in RCTs (Contentin et al., 2014). It seems a more defendable endpoint for trials than comparing ventilator in survivors and provides the greater statistical power to assess a treatment effect than the binary outcome measure of mortality (Yehya et al., 2019). Two large-scale and high-qualified RCTs (Kawazoe et al., 2017; Hughes et al., 2021) including in our meta-analysis both arrived at the same conclusion that the use of dexmedetomidine compared with propofol did not result in statistically significant improvement in 28/30-day mortality and ventilator-free days among septic patients who required mechanical ventilation.

We then compared the length of ICU stay between two groups. Prolonged length of ICU stay will increase the economic burden for patients. Patanwala et al. (Patanwala and Erstad, 2016) found dexmedetomidine use was associated with higher costs and increased lengths of ICU when compared with propofol for sedation. However, the author stated it was a retrospective study with the potential for unmeasured cofounders, thus future prospective trials are needed. The present meta suggested that the administration of dexmedetomidine reduced the length of ICU stay.

Several limitations of the present study should be considered. Firstly, although the sample size of our study is much larger than that of previous studies, more large-scale, multicenter, and high-qualified RCTs are essentially needed. Secondly, in the study conducted by Kawazoe et al. (2017), we couldn’t get entire information on whether all participants in the control group received propofol, and this study including patients on invasive and non-invasive mechanical ventilation might bias the outcome. Thirdly, few trials reported long-term endpoints like 90-day mortality, and criteria for sepsis or septic shock were not consistent, which may cause clinical heterogeneity. Last but not least, some data in the present meta-analysis were described as medians in the interquartile range, and we used a widely accepted method to estimate the sample mean and variance, which was considered reasonable by us.

## 5 Conclusion

In summary, the results of our meta-analysis suggested that administration of dexmedetomidine for sedation in septic patients who required mechanical ventilation was not associated with 28/30-day mortality and ventilator-free days, but it reduced the length of ICU stay. Due to the limitations of the sample sizes, further large-scale and high-qualified RCTs are urgently needed to confirm our findings.
